# On the wings of Icarus – the need for transcendence in addictive diseases

**DOI:** 10.3389/fpsyt.2025.1563871

**Published:** 2025-03-21

**Authors:** Human-Friedrich Unterrainer

**Affiliations:** ^1^ Faculty of Psychotherapy Science, Sigmund Freud Private University, Vienna, Austria; ^2^ Center for Integrative Addiction Research (CIAR), Grüner Kreis Association, Vienna, Austria; ^3^ Department of Religious Studies, University of Vienna, Vienna, Austria; ^4^ University Department of Psychiatry and Psychotherapeutic Medicine, Medical University of Graz, Graz, Austria

**Keywords:** addiction, attachment, emotion regulation, Greek mythology, narcissism, oceanic feelings, spirituality, transcendence

## Abstract

This essay employs the Icarus myth as a metaphor to examine the role of transcendence in addiction. Icarus’ disregard for boundaries, driven by his quest for transcendence, mirrors the behavior of individuals with addictive tendencies. Addiction, a chronic disorder marked by compulsive substance use or behaviors, is characterized by loss of control, tolerance, withdrawal, and life disruptions. Furthermore it is linked to traits such as impulsivity and sensation-seeking. Transcendence, the pursuit of experiences beyond ordinary limits, often manifests in addiction as a distorted attempt to address spiritual or existential voids. While practices like meditation can facilitate healthy transcendence (e.g., by influencing the periaqueductal grey activity), addiction provides only fleeting euphoria, perpetuating dependency. Sigmund Freud’s concept of “oceanic feelings” (discussed in correspondence with Romain Rolland) and Abraham Maslow’s notion of “peak experiences” parallel the addict’s pursuit of unity and self-actualization. However, addiction undermines genuine fulfillment and growth. Spirituality emerges as a pivotal factor in both the development and recovery of addiction, offering reconnection to meaning, purpose, and a higher power. Neuroscientific insights suggest ancient brain regions, such as the Periaqueductal Grey, may underlie the human drive for transcendence. Drawing on Jungian psychology, the essay highlights spirituality’s role in addressing existential crises and guiding recovery, echoing Jung’s “Spiritus contra Spiritum” as a principle for overcoming addiction. In conclusion, the essay advocates for a balanced approach in addiction therapy, harmonizing the innate human desire for transcendence with sustainable personal growth, and avoiding the extremes symbolized by Icarus’ fatal pursuit.

## 1 Introduction

The ancient Greek myth of Icarus parallels addictive diseases, where individuals fail to recognize limits or act without regard for consequences. It tells the story of Icarus and his father Daedalus, who escaped imprisonment in Crete using wings made of feathers and wax. Daedalus warned Icarus not to fly too high or low, but Icarus, overwhelmed by the freedom of flight, ignored the warnings. He flew too close to the sun, melting the wax, causing the wings to break. Icarus fell into the sea and drowned, while Daedalus, heartbroken, vowed never to fly again (1). Two key personality traits associated with addiction are impulsivity and sensation-seeking. Ersche et al. (2) studied 30 pairs of stimulant-dependent individuals and their non-addicted siblings, as well as a control group. They found that siblings of stimulant users exhibited higher impulsivity compared to the control group, while no significant difference was found for sensation-seeking. Stimulant-dependent individuals showed higher levels of both traits than their siblings and controls. The authors concluded that impulsivity may serve as a behavioral endophenotype for stimulant use disorder, while sensation-seeking could be an effect of chronic drug use. Applied to Icarus, this suggests a genetically predisposed, impulsive individual whose latent addictive tendencies (sensation-seeking) were triggered by the allure of sunlight—ultimately leading to self-destruction.

The concept of an “addictive personality” has been a topic of ongoing debate. However, evidence suggests that no single, distinct personality type predisposes individuals to addiction (3). Instead, vulnerability to drug addiction is influenced by a complex interplay of genetic, environmental, and psychological factors. Nevertheless, studies indicate a higher prevalence of borderline personality traits among individuals dependent on opioids. This notion is supported by our research, which identified significantly elevated levels of borderline personality organization and attachment dysfunctioning in various groups of substance-dependent patients compared to a healthy control group (4). Furthermore, recent findings suggest that disorganized attachment, in particular, may be associated with increased opiate use (5). Additionally, the literature highlights that personality traits, such as sensation-seeking and impulsivity, may play diverse roles in the development, progression, and treatment outcomes of addictive disorders (6). Conversely, individuals with personality disorders may turn to substances as a way of self-medication to cope with emotional pain or interpersonal difficulties. Genetic predispositions, early life trauma, and environmental stressors can contribute to both addiction and personality disorders, making their relationship more intertwined than isolated (7).

However, returning to the figure of Icarus, substance use can also be associated with exaggerated self-confidence or heightened narcissism. Addictive behavior as an expression of narcissistic incompetence is discussed in many places in the literature (8). The competitive relationship with the overwhelming father figure of Daedalus may have literally inspired the fatal change of course toward the sunlight. Icarus’ flight toward the sun, despite warnings, reflects the human tendency to rebel against boundaries or limitations, driven by unconscious desires and a yearning for transcendence (see (9) for further discussion). Addiction also means not accepting one’s own limits or exceeding them without knowing what awaits on the other side of the border. Through addictive behavior, a transcendent space of perception is sought, access to which might remain unattainable by other means (10).

## 2 The role of transcendence in addictive diseases

Addictive disorders, also referred to as dependency disorders, are chronic conditions characterized by a compulsive and uncontrollable urge to engage in certain substance use or behaviors. Substances associated with addiction include alcohol, drugs, nicotine, and medications, while behaviors such as gambling, excessive internet use, and work can also become addictive. The concept of addiction has been extensively studied and discussed. Broadly, an addictive disorder is defined by several key features: 1) Loss of control: The individual struggles to regulate substance use or behavior, persisting despite adverse consequences. 2) Development of tolerance: Over time, increasing amounts of the substance or intensification of the behavior are required to achieve the desired effect. 3) Withdrawal symptoms: Attempts to reduce or cease substance use or behavior often result in distressing physical or psychological symptoms. 4) Neglect of other life domains: Addiction consumes such a significant portion of the individual’s life that critical areas, such as work, relationships, or hobbies, are neglected.

Addiction can severely impact physical and mental health, social functioning, and overall quality of life. Professional treatment, incorporating medical, psychological, and social interventions, is often essential to achieve and maintain recovery, ultimately supporting a healthy, addiction-free life (see, for example (11),, for an in-depth discussion on the conceptualization of addiction).

Transcendence, derived from the Latin trans- (beyond) and scandare (to climb), refers to the concept of going beyond ordinary, material, or limited experiences. It signifies an understanding or experience that surpasses normal human perception or rational thought. In religious or spiritual contexts, transcendence involves rising above the physical world to connect with a higher power, the divine, or the universe. It can also refer to a sense of oneness or enlightenment that surpasses the mundane. Philosophically, transcendence explores the nature of reality, consciousness, or existence, seeking deeper truths beyond human limitations. As a subjective experience, transcendence varies among individuals and can be achieved through meditation, prayer, mystical experiences, artistic creation, or self-reflection (12).

William James, in his seminal work The Varieties of Religious Experience (1902), explored transcendence as a central aspect of human spiritual and religious life. He described transcendence as experiences that go beyond ordinary human consciousness, offering a connection to something greater than oneself—be it God, the universe, or a universal truth. James characterized these experiences as ineffable, noetic (providing profound insights), transient, and often accompanied by a sense of unity and peace. He viewed them as deeply personal yet transformative, emphasizing their capacity to inspire moral growth, creativity, and a sense of meaning. For James, transcendence was a legitimate dimension of human experience, bridging the psychological and the mystical (13).

## 3 The deep blue of the ocean and the dark swamp of addiction

The oceanic feeling, a concept explored through correspondence between Romain Rolland and Sigmund Freud, denotes a profound sense of unity and boundlessness. Rolland described it as a spontaneous, renewing experience transcending organized religion and forming the basis of all spiritual beliefs. Freud, however, interpreted it as a regression to an infantile state of unity with the mother, linking it to early narcissistic desires and rejecting its intrinsic religious meaning. The sea, as a metaphor for the oceanic feeling, embodies duality—discovery and risk, awe and fear. Romantic thinkers like Jules Michelet celebrated its inspirational power, while others, such as Herman Melville, highlighted its perilous aspects (14).

Addiction, in the context of Abraham Maslow’s concept of peak experiences, can be seen as a distorted pursuit of transcendence and fulfillment. While Maslow described peak experiences as moments of intense joy, clarity, and self-actualization, addiction often mirrors this search for transcendence but through unhealthy means. The addict may temporarily experience a fleeting sense of euphoria or relief, similar to the peak moments Maslow outlined, but this high ultimately leads to a cycle of dependency and suffering. Instead of fostering genuine personal growth, addiction narrows the individual’s sense of self and undermines long-term fulfillment, preventing true self-actualization (15).

After the sunlight robbed him of his feathers, Icarus found his grave in the infinite expanse of the sea or was swallowed up by it. The transcendent power of the sea as tremendum et fascinans (Latin for “fearful and fascinating,” describing the experience of the divine or the sacred (16)) is expressed here in a special way. Accordingly, oceanic feelings can simultaneously mean the sensation of dissolution in a greater whole or the experience of complete fragmentation. A state that can be induced by drug use is frequently found in literature. For example, Charles Baudelaire described entering an “artificial paradise” through the consumption of marijuana (17), while Aldous Huxley spoke of opening the “doors of perception” to a transcendental space through the consumption of LSD (18).

## 4 Spirituality as a neuro-affective state of mind

In Affective Neuroscience theory, seven basic or primary emotions—four positive: SEEKING, CARE, PLAY, LUST, and three negative: FEAR, ANGER, SADNESS—are assumed to originate in the brainstem and activate regions in higher levels of the cortex, particularly the limbic system and neocortex (19). Within this framework, the Periaqueductal Gray (PAG) has emerged as an area of special interest, as it is believed to be where primary process emotions originate. Recent research has shown that lesions in the PAG can decrease the capacity for spiritual experiences, nurturing the hypothesis that spirituality or related emotions may be anchored in this region. This finding enriches the canon of primary emotions, suggesting that the genesis of higher emotions, such as spirituality, might arise from ancient areas of the brain (20).

Jaak Panksepp, (who coined the term “Affective Neuroscience”), viewed spirituality as an important higher emotion, though he did not extensively elaborate on this concept. This created a research gap that our group has recently addressed by developing a psychometric instrument to assess oceanic feelings rooted in the PAG (21). In line with Jungian psychology, some argue that deep within the PAG lies the manifestation of the collective subconscious in the mammalian brain. This connection may explain why the need for transcendence is seemingly universal across humanity (22). Although direct studies on meditation’s effects on the PAG in the context of substance use disorders (SUD) are limited, the PAG’s role in pain modulation, emotional regulation, and addiction suggests that meditation may influence this region, potentially supporting SUD treatment (23).

The phrase Spiritus contra Spiritum (Latin for “spirit against spirit,” where spiritus denotes both “alcoholic spirit” and “divine spirit”) was emphasized by Carl G. Jung and by Bill Wilson, co-founder of Alcoholics Anonymous, in their correspondence. It encapsulates the tension between substance dependency and the human longing for spiritual fulfillment, illustrating the inner struggle between addiction and transcendence (24).

## 5 Concluding remarks

Spirituality in addictive diseases refers to the role of spiritual beliefs and practices in both the development and recovery from addiction. Many individuals struggling with addiction experience a spiritual void, often seeking fulfillment through substances or behaviors. Recovery, however, can involve reconnecting to a higher power, personal meaning, or purpose. Spirituality fosters inner peace, self-awareness, and a sense of belonging, enhancing resilience and supporting long-term sobriety (25). It addresses the emotional, psychological, and existential aspects of addiction, helping individuals find hope and healing beyond the physical symptoms of the disease (26, 27).

The myth of Icarus is often interpreted as a warning against hubris and overconfidence, illustrating the dangers of exceeding the limits of nature and reason. Icarus, in his quest to approach the sun (against the will of his father), crossed this boundary, paying the price for his recklessness with his life (28). Similarly, addiction therapy often involves guiding individuals out of the “dark night of the soul” caused by addiction and into the bright light of recovery. However, the intensity of that light can sometimes be overwhelming. Reflecting William Blake’s words, “some are born to sweet delight, others to endless night,” (29) it may be more beneficial for some individuals in recovery to remain in the penumbra—the softer, more manageable light—rather than striving for the full, blinding sun. Recovery, like the myth of Icarus, requires balance, humility, and a respect for one’s own limitations (30). The clinical implications of this metaphor are twofold. Firstly, addiction recovery necessitates a balanced therapeutic approach, where clinicians support individuals in making gradual, sustainable progress rather than striving for rapid or idealized outcomes. Secondly, emphasizing achievable goals and acknowledging patient limitations can mitigate the risk of overwhelm, enhancing the likelihood of long-term recovery.
